# An Objective System for Quantitative Assessment of Television Viewing Among Children (Family Level Assessment of Screen Use in the Home-Television): System Development Study

**DOI:** 10.2196/33569

**Published:** 2022-03-24

**Authors:** Anil Kumar Vadathya, Salma Musaad, Alicia Beltran, Oriana Perez, Leo Meister, Tom Baranowski, Sheryl O Hughes, Jason A Mendoza, Ashutosh Sabharwal, Ashok Veeraraghavan, Teresia O'Connor

**Affiliations:** 1 Department of Electrical & Computer Engineering Rice University Houston, TX United States; 2 Agricultural Research Service, US Department of Agriculture Children’s Nutrition Research Center Baylor College of Medicine Houston, TX United States; 3 Public Health Sciences Division Fred Hutchinson Cancer Research Center Seattle, WA United States; 4 General Pediatrics Department of Pediatrics University of Washington Seattle, WA United States

**Keywords:** television, screen media, digital media, measurement, child, gaze, machine learning, mobile phone

## Abstract

**Background:**

Television viewing among children is associated with developmental and health outcomes, yet measurement techniques for television viewing are prone to errors, biases, or both.

**Objective:**

This study aims to develop a system to objectively and passively measure children’s television viewing time.

**Methods:**

The Family Level Assessment of Screen Use in the Home-Television (FLASH-TV) system includes three sequential algorithms applied to video data collected in front of a television screen: face detection, face verification, and gaze estimation. A total of 21 families of diverse race and ethnicity were enrolled in 1 of 4 design studies to train the algorithms and provide proof of concept testing for the integrated FLASH-TV system. Video data were collected from each family in a laboratory mimicking a living room or in the child’s home. Staff coded the video data for the target child as the gold standard. The accuracy, sensitivity, specificity, positive predictive value, and negative predictive value were calculated for each algorithm, as compared with the gold standard. Prevalence and biased adjusted *κ* scores and an intraclass correlation using a generalized linear mixed model compared FLASH-TV’s estimation of television viewing duration to the gold standard.

**Results:**

FLASH-TV demonstrated high sensitivity for detecting faces (95.5%-97.9%) and performed well on face verification when the child’s gaze was on the television. Each of the metrics for estimating the child’s gaze on the screen was moderate to good (range: 55.1% negative predictive value to 91.2% specificity). When combining the 3 sequential steps, FLASH-TV estimation of the child’s screen viewing was overall good, with an intraclass correlation for an overall time watching television of 0.725 across conditions.

**Conclusions:**

FLASH-TV offers a critical step forward in improving the assessment of children’s television viewing.

## Introduction

### Television Viewing and Other Screen Use Among Youth

The American Academy of Pediatrics Council on Communications and Media has reported that children spend more time using screen media (television, movies, smartphones, tablets, computers, etc) than time in school [1]. Data from the Kaiser Family Foundation for the United States found children aged 8-18 years spend about 7.5 hours using screen media on a typical day, with some of the screen exposure involving multitasking several screens [2]. Nationally representative data of US children in 2020 found that children aged 5 to 8 years use an average of 3 hours and 5 minutes of screen media daily [3]. The types of screens children use have changed over the last decade [3,4]. Web-based videos, subscription streaming services, and television account for 73% of screen media use by children aged <8 years [3]. Similarly, in 2015, 62% of youths aged 9-12 years reported they watched television every day and television viewing remained one of the media activities enjoyed the most by tweens [5]. Television viewing therefore remains an important component of children’s overall screen use, which has been linked to detrimental cognitive development [6], worse child psychosocial outcomes [7], lower school achievement [7], child obesity [7,8], cardiometabolic risk [7], and decreased fitness [8]. Thus, higher levels of screen media use is a public health concern [9].

### Measuring Television Viewing Among Youth

Unfortunately, current methods to assess children’s television viewing and other screen media use remain inadequate, making it unclear how accurate television and screen media exposure estimates are. Tools are needed to objectively measure children’s use of screens across screen media platforms to ultimately inform a composite measure of *screen use*. New tools to track people’s screen use on mobile devices rely on background apps that record smartphone use [10,11] or obtain intermittent automatic screenshots of the mobile device to record how it is being used over a specific period [12]. Although both are important contributions to improve the assessment of children’s screen media use, they do not account for exposure to larger screens, such as televisions, computers, and stationary video game consoles. The risk of obesity differs based on the type of screen media used by a child [13,14], highlighting the importance of measuring all forms of screen media exposure. Although children’s screen media use is rapidly evolving with the use of many different devices and multiple web-based platforms for viewing content [15], television viewing is still a prominent behavior among youth [1,3,5,16].

The current gold standard to measure children’s screen use is direct or video-recorded observations that allow coding of the time a child spends watching a television screen [17-19]. Although accurate, this is too expensive and intrusive for most research studies, especially in-home settings. Most previous studies have relied on subjective recall by youth or their parents to assess television viewing and screen use [2,4,11,17]. This subjective assessment is prone to many sources of bias and errors, resulting in low accuracy estimates [18]. The most common method, self-reported or parent proxy-reported surveys of television viewing behaviors [17,19], has rarely been compared with a gold standard. Those that have, did not perform well. Anderson et al [18] compared parent-reported television diaries and general estimates of television viewing to the gold standard in a child’s home over the same period. Parent completed television diaries correlated moderately well with coded video observations (*r*_91_=0.67-0.86; *P*<.001). However, the correlation between parent estimates and coded video observations was significantly weaker (*r*_92_=0.27; *P*<.01) [18]. Furthermore, there was significant sample selection bias of families willing to participate in the study involving a high participant burden (television diaries), biased toward White, middle-class, and 2-parent households.

Objective automatic or passive methods for measuring children’s television viewing and use of other large stationary screens (computers and videogame systems) are needed to better assess children’s typical screen viewing and use behaviors. In the future, objective assessment of television viewing could be added to output from assessment tools of other screen platforms, such as mobile devices [11], for a composite measure of screen use among children. We are therefore developing an objective and automatic system to measure television viewing to allow a more comprehensive and accurate assessment of children’s television viewing to inform the assessment of screen media exposure. This paper describes the design and development of the resulting assessment tool, Family Level Assessment of Screen Use in the Home-Television (FLASH-TV), and the data acquired by the FLASH-TV system.

## Methods

### Ethics Approval

The Institutional Review Board at Baylor College of Medicine reviewed and approved the study protocol (H-40556).

### Overview of FLASH-TV Development

**Figure 1:**
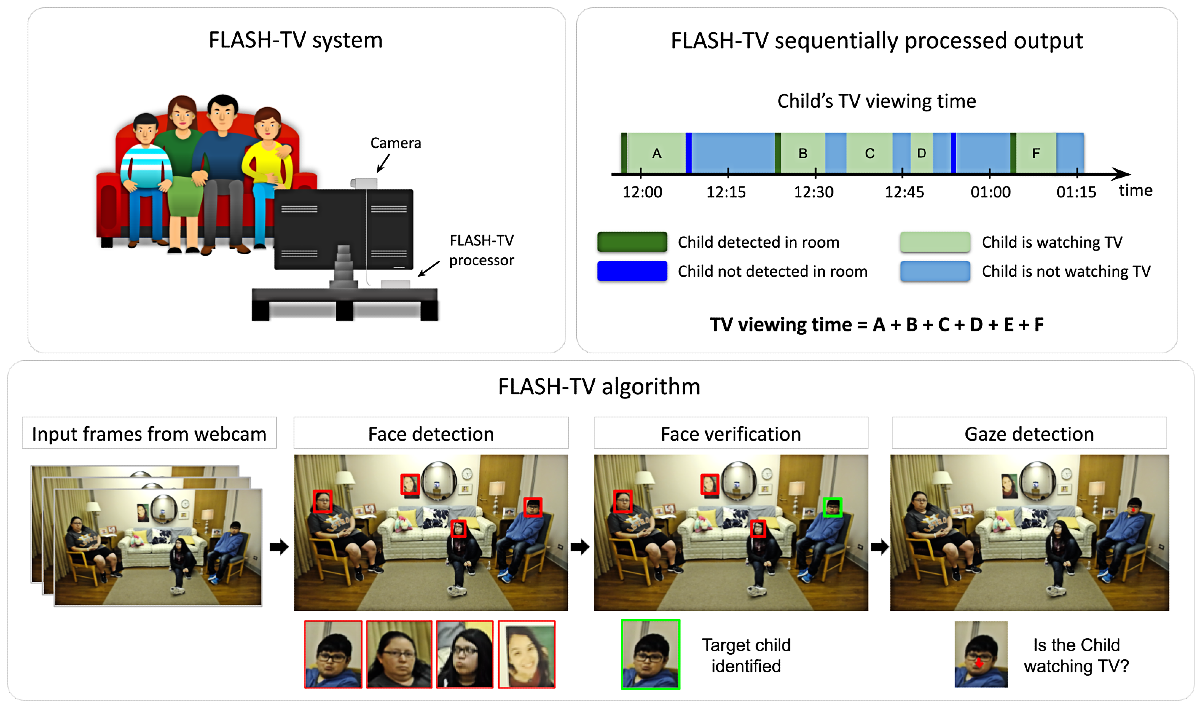
The integrated Family Level Assessment of Screen Use in the Home-Television (FLASH-TV) system. The FLASH-TV system takes as input video frames and processes it through a sequence of 3 steps: (1) Face detection, (2) Face verification, and (3) Gaze estimation to create a log containing a child’s television viewing time.

The overall goal of FLASH-TV is to estimate the total time a target child views a television or other large screen. To achieve this, FLASH-TV consists of a video camera (Logitech c930e 1080p) placed directly on or near the television, with the camera facing the viewers. The video camera records high-resolution images (approximately 1 megapixel or greater) at a rate of 15-30 frames/s. Computer vision and machine learning algorithms analyze each frame of the recorded video. Video analysis follows three stages: (1) *face detection—*to detect any faces present in every frame of the video, (2) *face verification—*to isolate and localize the presence of the target child in any frame, and (3) *gaze estimation—*to determine whether the target child is looking at the television (Figure 1).

### Data Collection Using the FLASH-TV System

Four small, iterative design tests were conducted to obtain video data to develop, train, and test the 3 steps required for a robust FLASH-TV. Three of the design tests were conducted in an observation laboratory at Baylor College of Medicine, which was set up as a living room. One of the design tests was conducted at the family’s home to test the system under natural circumstances. A parent and 2 siblings (one who was identified as the target child) were invited to participate in the task-based protocols. Inclusion criteria for each parent–sibling triad were: parent or legal guardian of children; target child aged 6-11 years and sibling aged 6-14 years; family fluent in English; and parents willing to allow their children to watch age-appropriate television or movies and play age-appropriate digital games. Exclusion criteria were: parent or child with developmental, medical, mental, or physical diagnosis that would prevent him or her from following the protocol. The research protocol was reviewed and approved by the institutional review board at Baylor College of Medicine with institutional review board reciprocity by Rice University via an established authorized agreement. All methods were performed in accordance with the Declaration of Helsinki and according to the federal and institutional guidelines. Informed written consent was provided by the parents of each triad for participation in the study and assent provided by all the children who participated. Participants in all design studies were offered an opt-in on the consent form to have their images used in reports and presentations that describe the development of FLASH-TV. All parents and children depicted in this document opted-in and additionally provided consent for their images to be used in publications by reviewing and signing the Baylor College of Medicine, media release form. Of the 22 participating families, one triad’s video data from design test 2 was corrupt. Here, we present the data from the remaining 21 triads.

Each design test protocol lasted approximately 90 minutes and contained minor variations. Each protocol required participants to watch television, engage with a mobile tablet, or play with physical toys while being video recorded by the observation room cameras as well as the prototype FLASH-TV system. Participants were asked to change their positions in the room (eg, from the couch to the floor) while performing each task for a few minutes at a time. For certain protocol segments, participants were asked to leave the room for a short period to ensure that FLASH-TV would detect their absence and return. The lighting of the room was varied for some tasks during several of the design tests to assess the robustness of FLASH-TV under bright, dim, and dark conditions. Each protocol included a 20- to 30-minute free-play portion to capture naturalistic viewing of a television screen by children when toys and a mobile device were also available. The room set up varied for each family, including different locations of the television and chairs in room and different room decorations. Design test 3 differed from design tests 1 and 2 as it included 2 separate, approximately 30-minute visits, 1 week apart from the observation laboratory so that the face verification could be assessed with participants across days. Design test 4 was conducted at the family’s home using a slightly modified protocol. An example of task-based protocols is provided in Multimedia Appendix 1.

FLASH-TV consisted of a high-definition, wide-angle video camera (Logitech 1080p webcam running at 15-30 frames/s) placed on top of the stationary screen (large computer monitor in the laboratory observation room or a television at the family’s home). The FLASH-TV video data from each of the design tests were reviewed and coded by trained behavioral research staff to determine whether the child was watching television. The research staff coded video data (available at the frame level) was considered the *gold standard* for training and testing the FLASH-TV machine learning algorithms. The target child was identified in each video frame, and then coded with one of four codes for the target child: watching screen, not watching screen, out of frame, or cannot tell using duration coding (one code was applied to the video data and remained until the child’s behavior changed). Eight research staff were trained and certified to correctly label gaze or no gaze ≥90% of the time. Overall, 10% of each family’s video data were double coded by 2 independent staff to determine interrater reliability (*κ*=0.88 with an SD 0.23 for laboratory observations; *κ*=0.83 with an SD 0.25 for in-home observations).

### Face Detection

YOLOv2 [20], a state-of-the-art convolutional neural network (CNN) originally proposed for object detection, was modified to develop the face detection component of FLASH-TV, using a publicly available code base [21]. The modification was based on a transfer learning paradigm in which previously learned model information from the YOLOv2 system was refined and adapted to the FLASH-TV context. YOLOv2 CNN was originally trained to detect common objects (eg, cars, humans, traffic lights, and animals), but was adapted for the FLASH-TV face detector to extract the parents’, siblings’, and target children’s faces from design test videos. We retained the first 16 layers of the original YOLOv2 model, whereas all the YOLOv2 layers after layer 16 were replaced with our own convolution and detection layers. The entire network was retuned using large-scale public facial data sets [22-24] to refine the FLASH-TV face detector. The FLASH-TV face detector returned *bounding boxes* with the 2D spatial coordinates around all detected faces in each video frame, as shown in the second box from the left in Figure 1.

**Figure 2:**
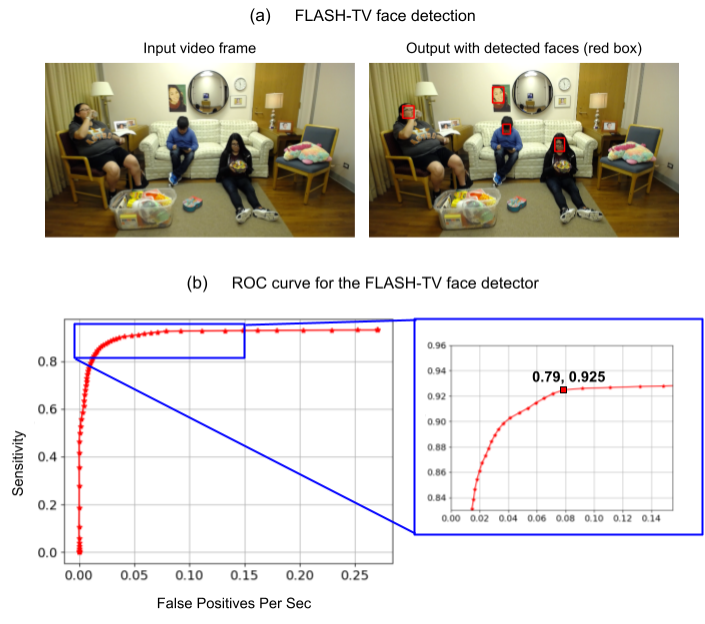
Family Level Assessment of Screen Use in the Home-Television (FLASH-TV) face detection. (a) FLASH-TV face detector takes an input frame and detects faces in the image, shown in red boxes (b) Receiver operating curve (ROC) for the face detector. The region indicated in the blue box in the ROC is enhanced in an inset to show the selection of the operating point. At the operating point, we have 0.79 false positives per second and a sensitivity of 92.5% to optimize false negatives.

A receiver operating curve analysis was performed on 10,000 test frames from design test triads 1, 2, and 3, stratified according to the task and lighting conditions to identify the threshold for the face detector. At the selected operating point, the false positive rate per second was 0.79, and the sensitivity was 92.5%. The goal was to set the face detector threshold in a range to avoid missing faces (false negatives) in exchange for accepting higher false positives. A false positive rate of 0.79 per second could be tolerated because most of these false positives would be screened out during the next stage of processing (the face verification step; Figure 2). In practice, about 96% of the false positives were screened out by face verification, achieving an effective false positive rate of 0.03 false positive face detections per second.

### Face Verification

The goal of FLASH-TV face verification was to determine whether any of the detected faces corresponded to the target child. DeepFace [25], a state-of-the-art method for face verification, was used to learn the face-specific features for face verification using deep neural networks with residual connections. A publicly available implementation of this approach from FaceNet [26,27] was trained for face verification on a publicly available data set, VGGFace2 [28], consisting of 3.3 million faces of 9000 identities. The resulting algorithm was tested on the Labeled Faces in the Wild test set [29] consisting of 5749 celebrities that were divided into 6000 face pairs, and DeepFace accuracy on this data set was 99.6%.

**Figure 3:**
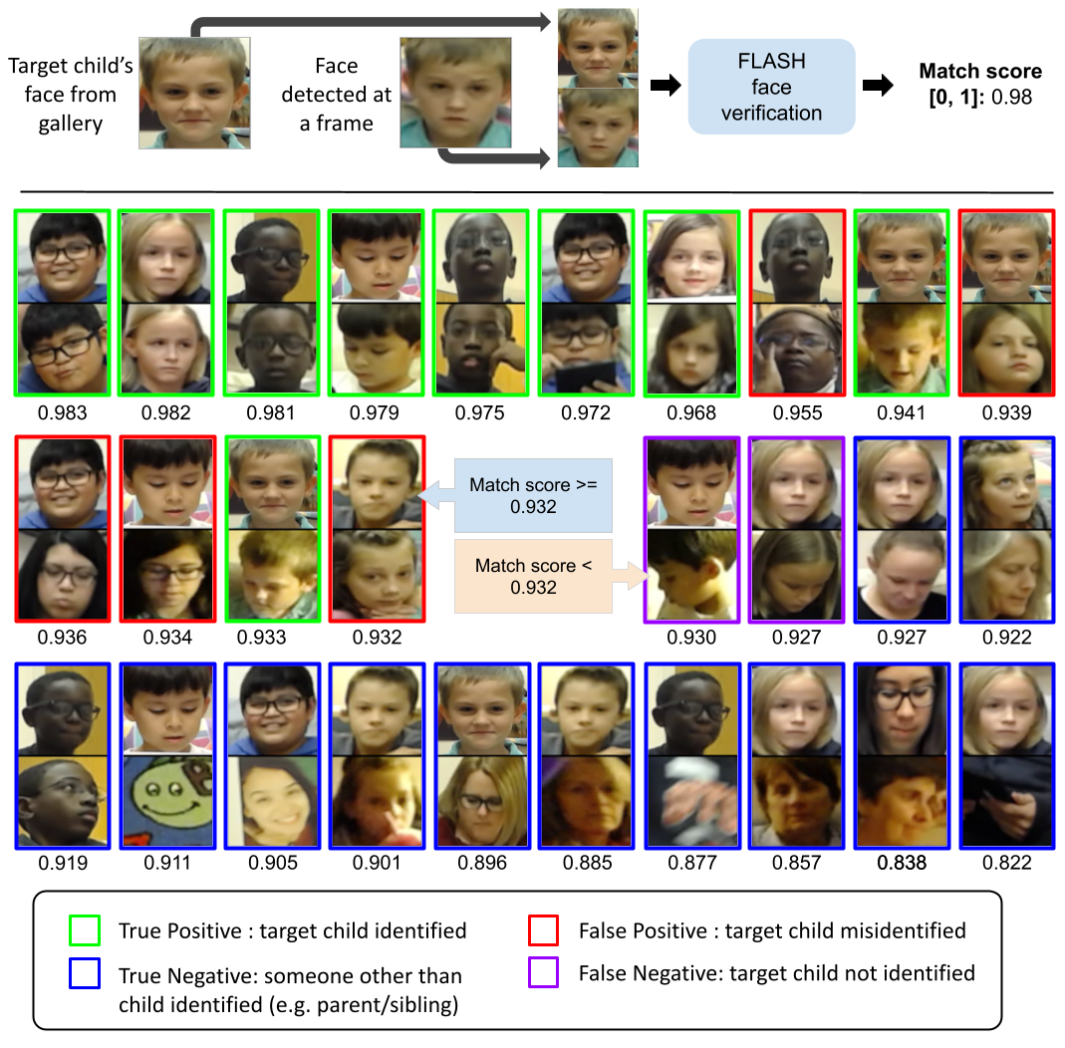
Family Level Assessment of Screen Use in the Home-Television (FLASH-TV) face verification. Demonstration of the FLASH-TV face verification approach. All faces identified in the face detection step are compared with a series of images of the target child’s face obtained at the start of the study protocol. The FLASH-TV algorithm assigns a similarity score of each face identified to an image of the target’s child’s face. At a match score threshold of 0.932, we optimize our true positives and true negatives compared with staff coding of faces among a random sample of 6000 pairs of faces with half pairs being similar faces. Face pairs with match scores above the threshold are considered as similar faces and below that as dissimilar faces. We show example face pairs with match scores in the range (0.991-0.822). Actual false positives and miss detection rates were much lower but we show several examples of each here simply for illustrative purposes.

To compute the similarity between the face in the bounding box (output of face detector) to the gallery of images of the target child, the correlation among their FaceNet features was measured. For design tests 1 and 2, approximately 33,000 randomly selected test frames were used, and for design test 3, approximately 4000 randomly selected test frames were used. As seen in Figure 3, the match score is closer to 1 when comparing the faces of the target child to another image of the target child. A match score threshold of 0.93 (identified by receiver operating curve analysis) was used in our implementation of FLASH-TV as it provided a reasonable trade-off between false positives and false negatives.

Preliminary analysis of face verification performance indicated that the low-light level with the resultant noisy image was the principal cause of face verification errors. Therefore, we refined the face verification model by retraining the system on a large data set of synthetic low-light, high-noise videos (where noise was added to existing video data to simulate low-light conditions). Further, we exploited the continuity of face identity across successive video frames by automatically tracking and smoothing identity evolution across frames. FLASH-TV face verification resulted in 93% accuracy in identifying the target child (see *Results* section for details).

### Gaze Detection

The goal of FLASH-TV gaze detection was to determine whether the target child was looking directly at the television (from which we inferred attention to the television). This was done by first detecting the target child’s eyes and then estimating their gaze direction (in relation to the location of the television). Prior gaze detection systems focused on estimating gaze direction from high-resolution images of eyes recorded on mobile phones, tablets, or laptops, where the distances were less than a meter [30-34]. Unfortunately, the FLASH-TV gaze detector had to work with the small facial image sizes (typically <50×50 pixels) captured in the bounding boxes from the video data as the subjects were farther away (2-4 m), and the camera had to cover a large field of view. Consequently, existing trained models for gaze estimation could not be directly used within the FLASH-TV context.

**Figure 4:**
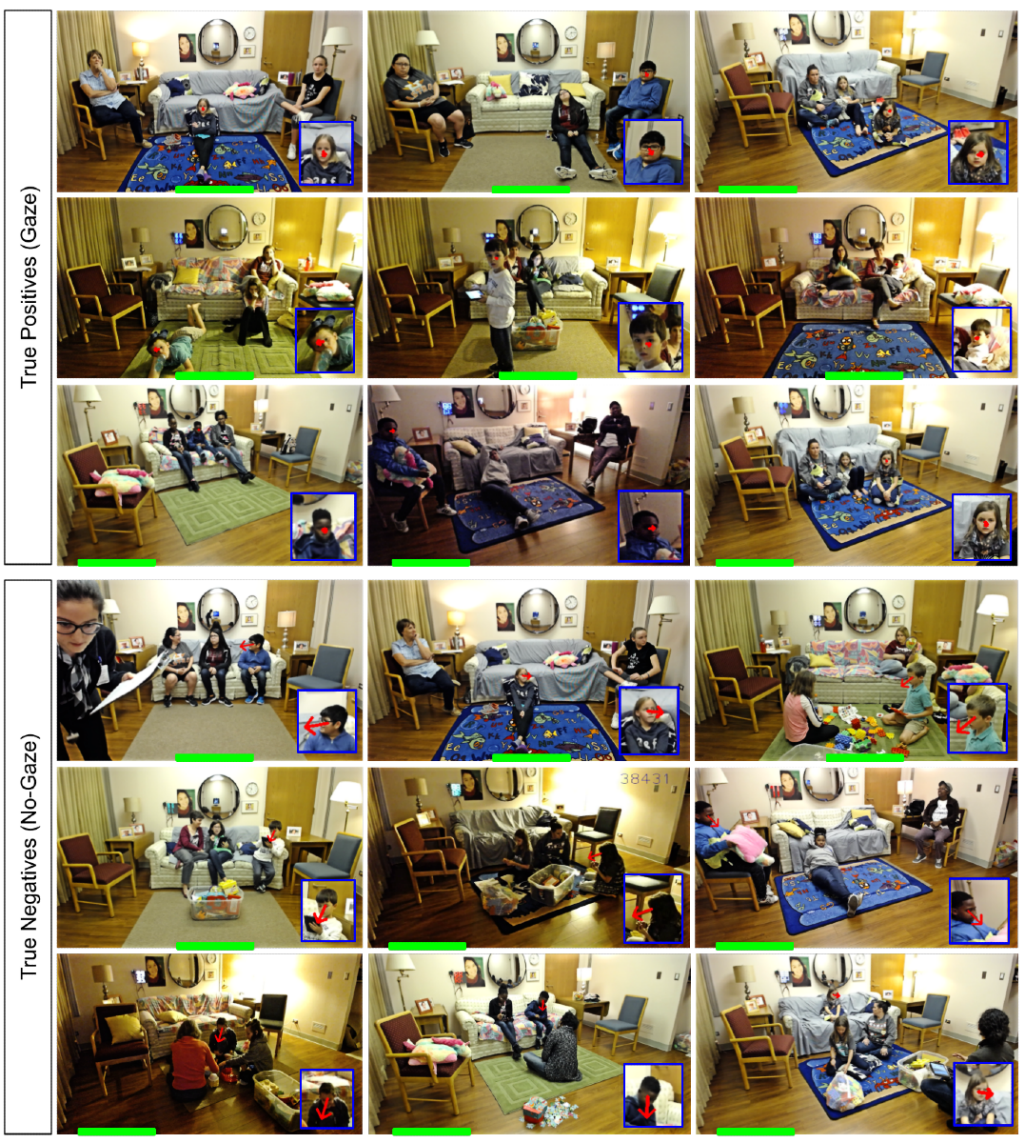
Family Level Assessment of Screen Use in the Home-Television (FLASH-TV) gaze estimation. All images identified as the target child by the FLASH-TV face verification step in a bounding box are processed for the direction of the child’s gaze based on Gaze360 algorithms [35]. This illustrates the resulting gaze vector (red arrow) that are classified as true positives (gaze) or true negatives (no gaze) by the system. Note, the angle of the gaze vector that is considered a true positive (gaze) will depend on the location of the television in the foreground. The approximate television location is indicated by a green box at the bottom of each image.

We adapted the Gaze360 approach of Kellnhofer et al [35] for FLASH-TV gaze estimation using a publicly available code base [36]. Gaze360 [35] provided a direction vector specifying the direction in which the person was looking (Figure 4). For FLASH-TV gaze detection, a dichotomous output, whether the child’s gaze was or was not on the television was desired. To obtain this dichotomous output from the gaze direction, angular limits were set on the direction of the vector, which should be identified as gaze, and outside, which should be no-gaze. These angular vector limits depended on where the face was in the frame and the relative position of the television (eg, notice the gaze directions for different locations in the video frame shown in Figure 4). To address this, the video frame was divided into multiple regions, for which we identified the angular limits for each. To account for the location of the television in the room, we labeled each FLASH-TV data set with relative position information between the FLASH-TV camera and the television. For example, of the 16 triads for design tests 1, 2, and 3, we have 10 triads with television in the center and 5 with television in the left. One family’s data were obtained from a unique position (below television) and could not be used in gaze estimation training or testing.

We used the leave-one-out strategy to evaluate the Gaze360 on FLASH-TV data. For each design test, we removed one of the family triads as test data and used the remaining families’ video data to train the algorithm. This was repeated for each triad in the design tests. The training data were used to obtain the angular limits for the gaze vector, which were then applied to the test data, resulting in a dichotomous gaze or no-gaze output. This binary output was compared with the gold standard human coding of the video data. Our FLASH-TV gaze detector achieved an accuracy of 87% (see the *Results* section for details).

At the end of each data collection session, the parents were asked about their perceptions of FLASH-TV using a structured interview guide. The brief interviews were audio-recorded, transcribed, and coded for themes by 2 trained staff members using NVivo (version 11, QSR International; 2015).

### Statistical Analysis

A summary of the algorithms used by FLASH-TV for face detection, face verification, and gaze estimation can be found in Table 1. For each individual step of video data processing, FLASH-TV output was compared with the gold standard (staff-coded video data), and the accuracy, sensitivity, specificity, positive predictive value (PPV), negative predictive value (NPV), false positives per second, and processing time were calculated within each family and then averaged across families (Table 2).

**Table 1:** Family Level Assessment of Screen Use in the Home-Television (FLASH-TV) algorithms.

FLASH-TV methods	Algorithm used	FPS^a^ processing
Face detection	Modified YoLo [20]	20
Face verification	FaceNet [26,27]	12
Gaze estimation	Gaze360 [35,36]	30

^a^FPS: frames per second.

**Table 2:** Outcome metrics assessed.

Outcome metric	Formula	Interpretation in reference to gaze
Accuracy	TP^a^+TN^b^/(TP+TN+FP^c^+FN^d^)	Overall how often does FLASH-TV^e^ make a correct prediction for gaze
Sensitivity	TP/(TP+FN)	High sensitivity indicates that when the child is watching television, FLASH-TV reports it as “Gaze” (few false negatives)
Specificity	TN/(TN+FP)	High specificity indicates that frames in which child is not watching television, FLASH-TV reports it as “no gaze” (few false positives)
PPV^f^	TP/(TP+FP)	High PPV indicates FLASH-TV “gaze” output corresponds to the child actually watching television
NPV^g^	TN/(TN+FN)	High NPV indicates FLASH-TV “no gaze” output corresponds to the child actually NOT watching television
FPR^h^	FP/(FP+TN)	High FPR corresponds to incorrectly identifying the child is watching television, when the child is actually NOT watching television

^a^TP: true positive (ie, FLASH-TV gaze agrees with the gold standard).

^b^TN: true negative (ie, FLASH-TV no gaze agrees with the gold standard.

^c^FP: false positive (ie, FLASH-TV gaze does not agree with the gold standard).

^d^FN: false negative (ie, FLASH-TV no gaze does not agree with the gold standard).

^e^FLASH-TV: Family Level Assessment of Screen Use in the Home-Television.

^f^PPV: positive predictive value.

^g^NPV: negative predictive value.

^h^FPR: false positive rate.

For face detection and face verification, the results were presented for the overall video data and stratified on whether the child’s gaze was on the television or not, as identified during the gold standard staff-coded video data. Stratifying by child gaze allows FLASH-TV to be evaluated in the context in which FLASH-TV needs to perform well, when the child is actually watching television, to estimate the target child’s screen viewing or use time. The robustness or reliability of the face verification to identify the target child across different days was assessed in design test 3, when the parent-sibling triad returned to the observation laboratory for a second data collection session about 1 week after the initial data collection. As the 2 visits were conducted on the same family, the average difference between visits in the outcome metrics (sensitivity, specificity, accuracy, PPV, NPV, and false positive rate) was calculated and tested using the nonparametric Wilcoxon signed-rank test.

To further assess the face verification across different days, exploratory generalized linear modeling was conducted to determine the difference in the outcome metrics (sensitivity, specificity, accuracy, PPV, NPV, and false-positive rate) by visit. A compound symmetry correlation structure was assigned to account for the nesting of repeated measurements within each family per visit (because of multiple frames per visit). A Poisson distribution was specified for all metrics except PPV, where a binomial was specified to fit the data. The effects of visit and family were tested as the main effects. The estimated difference in the response probabilities (least square means) of the outcome was obtained.

The goal of FLASH-TV was to estimate the target child’s television viewing time. The target child’s total television viewing time was estimated by sequentially running the 3 steps of FLASH-TV and summing the duration of time the child’s gaze was on the screen (given in minutes:seconds format). To assess the target child’s television viewing time estimated by the FLASH-TV system compared with the gold standard, the agreement between the number of frames identified as television viewing (after sequentially running each step) by the FLASH-TV was compared with staff codes using the prevalence and bias-adjusted *κ*. Moreover, reliability was assessed by means of the intraclass correlation coefficient (ICC) using a generalized linear mixed model accounting for the binary outcome (television viewed or not viewed). A random frame nested within the family effect was specified to reflect the ordering of the frames within family. Correlations of ≤0.35 were defined as weak, 0.36 to 0.67 as moderate, ≥0.68 as high, and ≥0.9 as very high [37]. The ICC was also used to determine the reliability of the target child’s total television viewing time estimated by the FLASH-TV system compared with the gold standard using a generalized linear mixed model specifying a lognormal distribution for the continuous outcome and random frame nested within the family. Data from the in-home data collection were used to independently test each algorithm step and then the sequential assessment of each step for the overall estimation of television viewing time for the child. Analyses were conducted using SAS (version 9.4, SAS Institute, Inc). Significance was determined using a two-sided *α* value of .05. Face recognition and verification algorithms can introduce potential race bias if the algorithm accuracy varies according to the race of the child [38]. Therefore, we report television viewing time estimates from FLASH-TV and the gold standard stratified by child race, but the small sample size precludes statistical comparisons.

## Results

### Overview

The demographics of the 21 parent-child triads (Table 3) indicate a racially and ethnically diverse sample of families took part in the design tests.

**Table 3:** Demographics.

				Overall		Design test 1		Design test 2	Design test 3 (2 visits)		Design test 4 (in-home)
Parent-sibling triads (n)				21		5		6	5		5
**Children, n (%)**				42 (100)		10 (100)		12 (100)	10 (100)		10 (100)
	Age (years), mean (SD)		10.2 (2.1)		10.1 (2.5)		9.9 (2.1)		10.5 (1.9)	10.5 (2.0)	
	Sex (female), n (%)		25 (57)		6 (60)		5 (42)		7 (70)	7 (70)	
	**Race and ethnicity, n (%)**										
		Non-Hispanic White	16 (38)		4 (40)		4 (33)		2 (20)	6 (60)	
		Hispanic White	8 (19)		2 (20)		0 (0)		4 (40)	2 (20)	
		Non-Hispanic Black	10 (24)		2 (20)		6 (50)		2 (20)	0 (0)	
		Hispanic Black	2 (5)		0 (0)		0 (0)		2 (20)	0 (0)	
		Asian	2 (5)		0 (0)		2 (17)		0 (0)	0 (0)	
		Other (mixed or Hispanic Other)	4 (10)		2 (20)		0 (0)		0 (0)	2 (20)	
**Parent, n (%)**				21 (100)		5 (100)		6 (100)	5 (100)		5 (100)
	Age (years), mean (SD)		43.9 (8.7)		42.6 (6.9)		46 (9.4)		43.8 (8.6)	42.8 (6.7)	
	Sex (female), n (%)		19 (91)		5 (100)		6 (100)		5 (100)	3 (60)	
	**Race and ethnicity, n (%)**										
		Non-Hispanic White	9 (43)		3 (60)		2 (33)		1 (20)	3 (60)	
		Hispanic White	4 (19)		1 (20)		0 (0)		2 (40)	1 (20)	
		Non-Hispanic Black	5 (24)		1 (20)		3 (50)		1 (20)	0 (0)	
		Hispanic Black	1 (5)		0 (0)		0 (0)		1 (20)	0 (0)	
		Asian	1 (5)		0 (0)		1 (17)		0 (0)	0 (0)	
		Other (mixed or Hispanic Other)	1 (5)		0 (0)		0 (0)		0 (0)	1 (20)	
	**Education, n (%)**										
		High school	2 (10)		0 (0)		0 (0)		1 (20)	1 (20)	
		Some college	6 (29)		2 (40)		1 (17)		2 (40)	1 (20)	
		College	9 (43)		3 (60)		3 (50)		1 (20)	2 (40)	
		Graduate school	4 (19)		0 (0)		2 (33)		1 (20)	1 (20)	

### Face Detection

**Figure 5:**
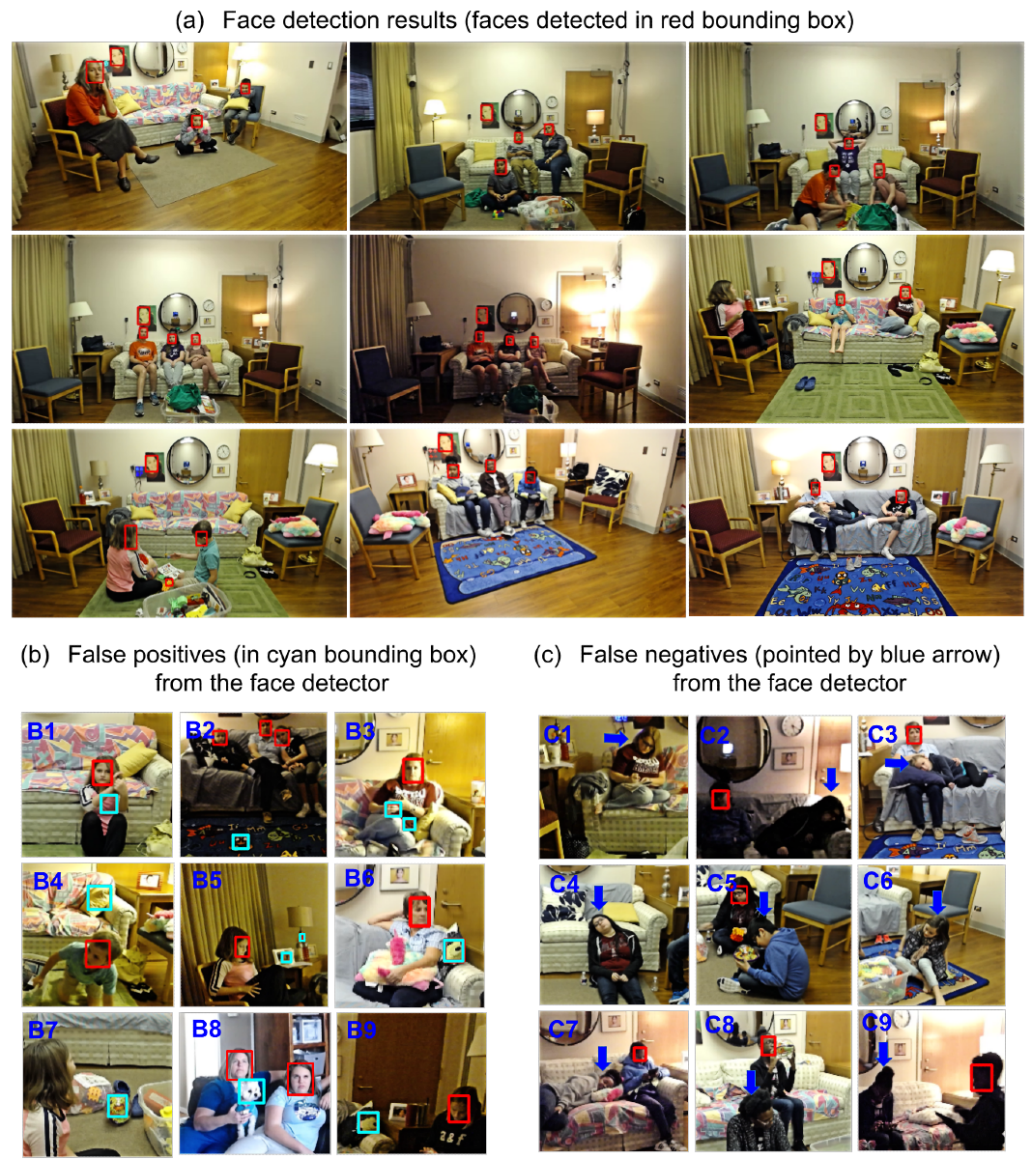
Face detection results. (a) Family Level Assessment of Screen Use in the Home-Television (FLASH-TV) face detector captures the faces (indicated in red boxes) across tasks and lighting conditions. (b) Examples of false positives (regions that are not human face) identified by FLASH-TV face detector. Notice the patterns identified as faces (B1, B3, B4, B6, B7, and B9); also the cartoon face detected (B2) and the animal face detected (B8). (c) Examples of false negatives (human faces that are not detected) by FLASH-TV face detector (lacking a red bounding box). The face detector has difficulty in detecting faces, when the faces are not orientated upright (C3, C4, and C7), when the face is partially occluded (C5, C6, and C8), and when the lighting is dark (C2 and C9).

Table 4 reports the outcomes for FLASH-TV face detection algorithm alone. The FLASH-TV face detector achieved a mean conditional (ie, when the child’s gaze was on the television) sensitivity of 95.5% (SD 4.79%) with 0.43 (SD 0.51) false positives per second for design tests 1 and 2 on approximately 33,000 test frames. For design test 3, the conditional sensitivity was 96.4% (SD 3.61%) with 0.2 (SD 0.06) false positives per second on approximately 4000 randomly selected test frames. The face detector was also tested with the in-home data from design test 4, which provided 7.5 hours of video data from 5 parent-sibling triads. The face detector’s conditional sensitivity was 97.9% (SD 0.02%) with 0.3 (SD 0.15) false positives per second on approximately randomly selected 20,000 test frames, supporting a high accuracy in real-life scenarios and providing greater confidence that the face detector is functioning at an appropriate accuracy to be used in the three-step process of estimating a child’s screen use on larger screens. Our current FLASH-TV face detector is running at 20 frames per second. Exploratory qualitative review of the false positives (regions that are not human face) identified by the FLASH-TV face detector included patterns in cushions and surroundings, cartoon faces, and animal faces (Figure 5). Examples of false negatives (human faces that are not detected) identified by FLASH-TV face detector (lacking a red bounding box) included instances when the faces were not oriented upright (eg, reclining on sofa), were partially occluded, or were in low-light settings.

**Table 4:** Family Level Assessment of Screen Use in the Home-Television face detection^a^.

		Sensitivity (%; range)	Positive predictive value (%; range)
**Design tests 1 and 2 (n=11 triads)**			
	Overall (target child, sibling, and parent)	91.9 (83.2-96.7)	86.6 (67.4-93.9)
	With gaze on television (target child)	95.5 (78.8-96.9)	74.9 (53.6-93.0)
	Without gaze on television (target child)	87.7 (78.8-96.9)	55.1 (26.8-73.0)
**Design test 3—two visits combined (n=5 triads)**			
	Overall (target child, sibling, and parent)	96.2 (93.4-98.7)	83.5 (72.3-89.8)
	With gaze on television (target child)	96.4 (87.5-100.0)	61.9 (38.4-73.4)
	Without gaze on television (target child)	89.8 (73.0-96.4)	48.1 (37.6-64.0)
**Design test 4—in-home observation (n=5 triads)**			
	Overall (target child, sibling, and parent)	92.0 (83.9-99.5)	70.1 (54.5-86.6)
	With gaze on television (target child)	97.9 (94.7-99.9)	52.5 (30.8-75.4)
	Without gaze on television (target child)	86.1 (65.2-97.5)	42.1 (30.7-71.7)

^a^True negatives are not meaningful to assess for face detection because they represent everything in the video that is not detected as a face. Therefore, accuracy, specificity, and negative predictive values (that depend on true negatives) were not calculated for face detection.

### Face Verification

**Figure 6:**
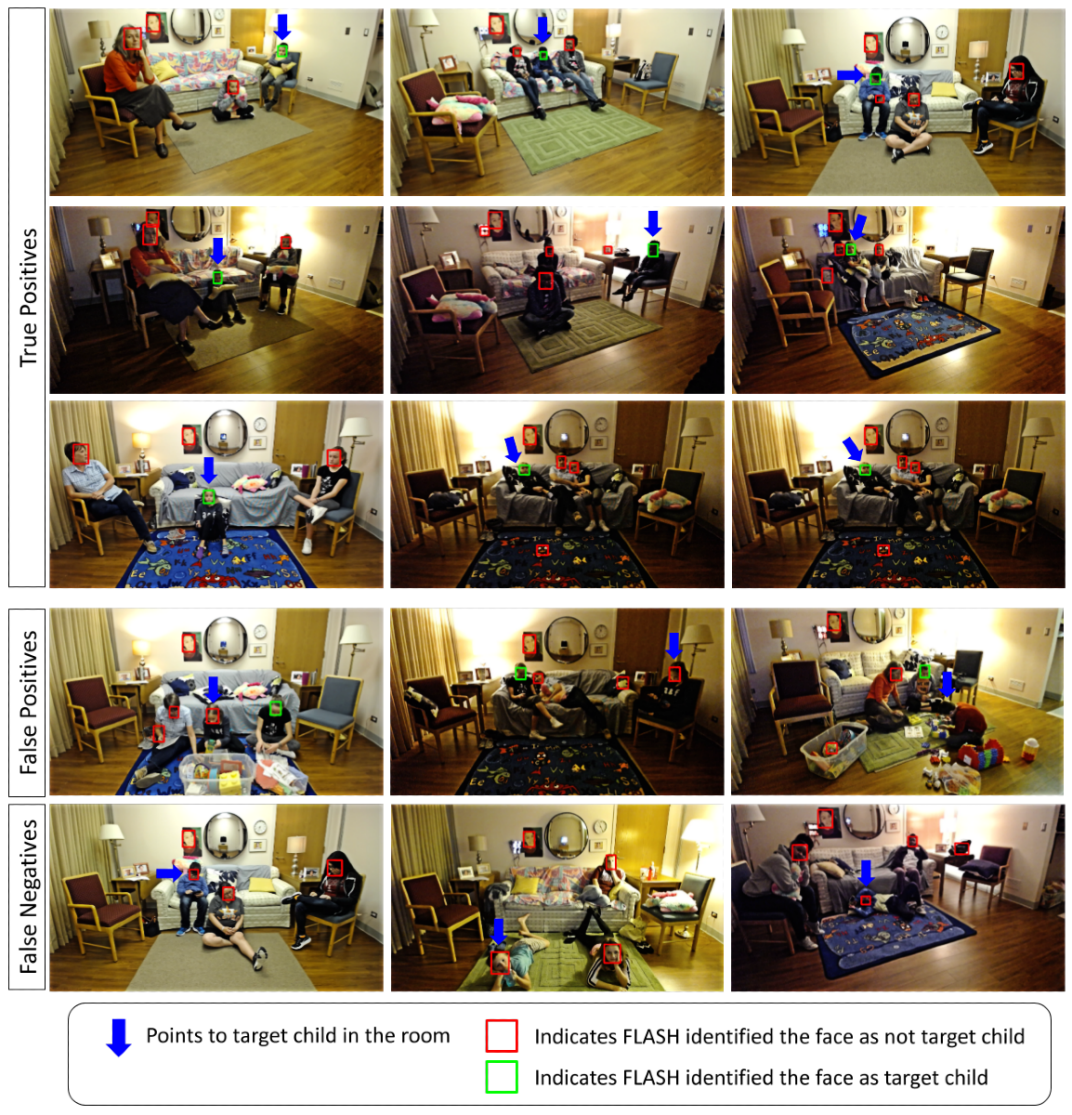
Family Level Assessment of Screen Use in the Home-Television (FLASH-TV) face verification results. This figure shows the frames from different participants where the FLASH-TV face verification identifies the target child in the frame. True positives indicate when the child is identified correctly. False positives indicate when a parent or sibling is mistaken as the target child and false negatives indicate when the target child is not identified correctly. Most of the errors occur when the target child’s face is partially occluded when they are not watching television and when the lighting in the room is dim. Note that if the target child’s face is not identified when there is no-gaze, it will not affect our television viewing time.

Table 5 reports the outcomes for FLASH-TV face verification algorithm alone. For design tests 1 and 2, our face verification method achieved a mean conditional (ie, when the child’s gaze was on the television) sensitivity of 93.1% (SD 7.03%) for identifying the target child on approximately randomly selected 33,000 test frames. For design test 3, a conditional sensitivity of 96.1% (SD 3.77%) was achieved on randomly selected approximately 4000 test frames. Similarly, on our in-home data set from design test 4, the sensitivity was 91.3% (SD 15.71%) for identifying the target child. The current speed of face verification is 12 frames per second. Examples of false positives and false negatives for face verification for the target child can be found in Figure 6. Exploratory qualitative review of the errors revealed these happened when the target child’s face was partially occluded when they were not watching television and when the lighting in the room was dim, similar to face detection. Note that not identifying the target child’s face when there is no-gaze will not affect our final television watching time.

Using the Wilcoxon signed-rank test to test the mean difference across visits 1 and 2 in design study 3, small differences in mean sensitivity (−0.05, SD 0.46), accuracy (−0.01, SD 0.18), and NPV (−0.01, SD 0.15) were identified, with mean values being lower in visit 2 than in visit 1. These differences were identified overall, and were no longer significant for times when the child’s gaze was on television for all outcomes except NPV. The generalized linear models showed that the outcome metrics (sensitivity, specificity, accuracy, PPV, NPV, and false positive rate) did not differ by visit. Mean differences in response probabilities for visit 2 relative to visit 1 for the sensitivity was 0.99 (*P*=.86), specificity 0.99 (*P*=.37), accuracy 0.99 (*P*=.93), PPV 0.87 (*P*=.48), NPV 1.0 (*P*=.87), and the false-positive rate 1.07 (*P*=.27). Mean differences did not change remarkably after stratifying by gaze status (not shown). However, large differences between family pairs (>20%) were observed only for PPV, specifically 23.32% (95% CI 16.71%-32.29%) and 44.41% (95% CI 32.77%-60.18%). These differences persisted only when the child was viewing television.

**Table 5:** Family Level Assessment of Screen Use in the Home-Television face verification of target child.

		Accuracy (%; range)^a^	Sensitivity (%; range)^a^	Specificity (%; range)^a^	Positive predictive value (%; range)^a^	Negative predictive value (%; range)^a^
**Design tests 1 and 2 (n=11 triads^a^)**						
	Overall	92.8 (83.6-96.8)	78.0 (59.2-92.3)	97.2 (88.6-99.6)	89.0 (65.0-98.8)	93.8 (88.9-97.7)
	With gaze on television	97.8 (94.6-99.6)	93.1 (77.1-98.9)	99.4 (96.9-100)	98.2 (90.5-100)	97.7 (93.4-99.6)
	Without gaze on television	91.2 (87.7-96.5)	71.9 (51.9-92.0)	96.6 (84.9-99.4)	85.9 (54.8-98.8)	92.4 (87.1-97.4)
**Design test 3—two visits combined (n=5 triads)**						
	Overall	94.5 (85.5-99.0)	83.7 (42.2-97.2)	97.3 (90.3-99.6)	89.7 (64.2-98.9)	95.84 (87.3-99.1)
	With gaze on television	96.1 (83.5-99.6)	96.1 (85.9-98.6)	96.2 (82.8-100)	89.5 (53.7-100)	98.08 (95.9-99.5)
	Without gaze on television	92.9 (84.7-98.7)	73.9 (24.6-98.2)	97.7 (90.2-100)	90.1 (65.5-100)	93.80 (84.6-99.4)
**Design test 4—in-home observation (n=5 triads)**						
	Overall	94.3 (82.6-99.1)	86.9 (66.3-98.7)	99.1 (98.5-99.75)	97.7 (93.9-99.5)	92.7 (73.8-99.4)
	With gaze on television	95.7 (81.3-99.9)	91.3 (63.4-99.9)	99.8 (99.5-100)	99.7 (99.3-100)	93.6 (72.6-99.5)
	Without gaze on television	91.2 (83.3-96.1)	79.7 (42.0-97.0)	96.8 (94.8-98.8)	92.3 (81.0-98.7)	89.9 (79.7-98.6)

^a^Data analyzed at the frame (ie, bounding box). Given the small sample sizes in each design test, the mean and range (minimum–maximum) are reported.

### Gaze Detection

**Figure 7:**
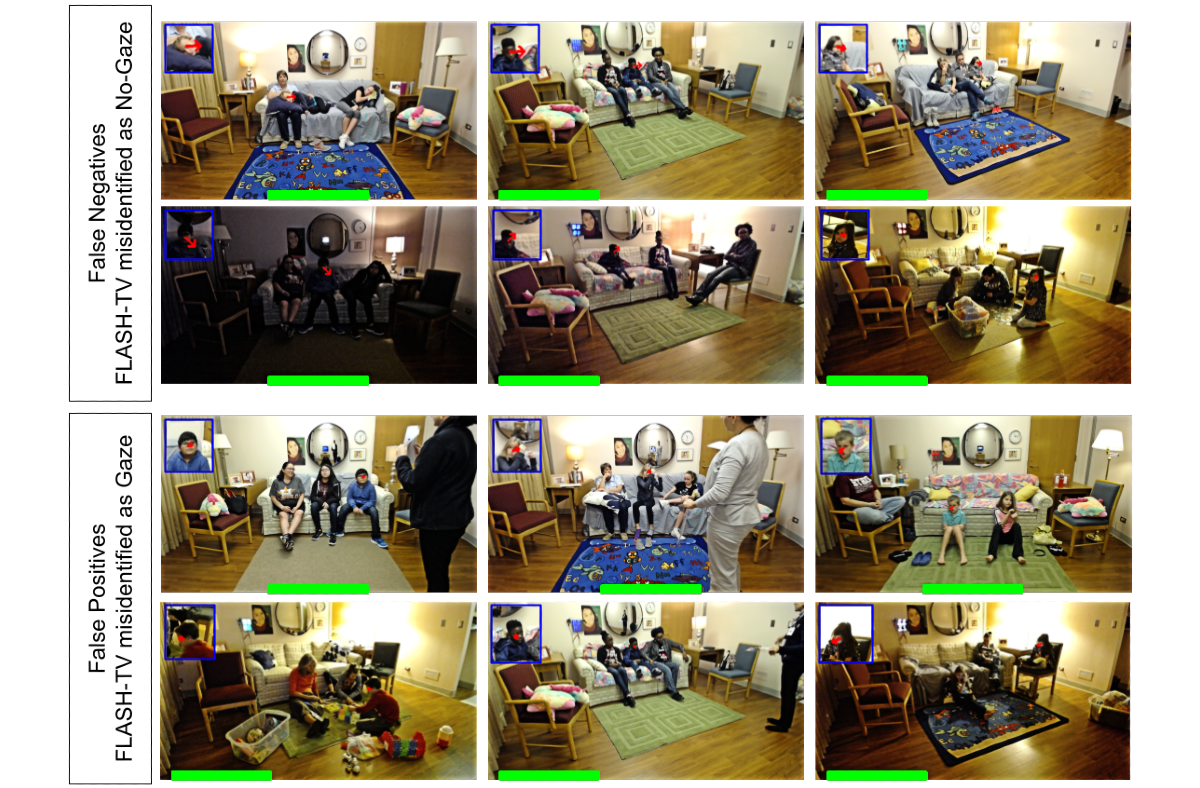
Family Level Assessment of Screen Use in the Home-Television (FLASH-TV) gaze estimation errors. This illustrates examples of errors from the FLASH-TV gaze estimator. The top two rows show false negatives resulting from FLASH-TV identifying no gaze on television, but staff coded gaze (gold standard). Qualitative assessment identified common reasons for false positives included low-light conditions (second row), the child’s face orientation not orientated upright (first row leftmost). Bottom two rows show false positives when FLASH-TV identified gaze, but the staff coded no gaze (gold standard). Qualitative assessment identified common reasons for false negatives included that gaze estimator has difficulty when children pay attention to something close to the television but not on the television (third row) and low-resolution (fourth row middle). The television location is indicated by a green box at the bottom of each image.

Table 6 reports the output for the gaze estimation algorithm alone. For gaze detection with television position in center (10 families), the mean accuracy was 87.2% (SD 7.38%) and mean sensitivity and specificity of 81% (SD 25.3%) and 86.8% (SD 7.14%), respectively (Table 5). For television position to the left of the room (5 families), the mean accuracy was 87% (SD 6.05%) and mean sensitivity and specificity of 76.2% (SD 20.9%) and 90.8% (SD 2.94), respectively. The current speed at which our gaze detection processes the frames is 30 frames per second. Figure 7 illustrates the most common errors for gaze estimation.

**Table 6:** Gaze detection of target child.

Television position	Accuracy (%; range)	Sensitivity (%; range)	Specificity (%; range)	Positive predicative value (%; range)	Negative predictive value (%; range)	False positives rate (%; range)
Center of wall (n=10)^a^	88.0 (74.1-93.1)	73.2 (27.8-95.3)	91.2 (75.8-96.9)	82.1 (68.1-93.7)	87.8 (75.3-98.1)	8.82 (3.1-24.3)
Left corner of room (n=5)^a,b^	87.1 (76.8-91.8)	76.2 (54.0-96.1)	90.8 (87.3-94.4)	74.2 (65.5-86.2)	91.5 (76.5-98.7)	9.2 (5.62-12.7)
In-home television position varied (n=5)^c^	75.6 (54.9-93.7)	73.4 (45.2-95.4)	82.7 (71.2-91.1)	90.8 (73.4-97.3)	55.1 (30.7-80.6)	17.3 (8.9-28.8)

^a^Design tests 1 to 3 (in observation laboratory data collection).

^b^One family’s data from design tests 1 to 3 were obtained from a unique position (below television) and could not be used in gaze estimation training or testing.

^c^Design test 4 (in-home data collection).

### Overall Television Viewing Time Estimation

**Figure 8:**
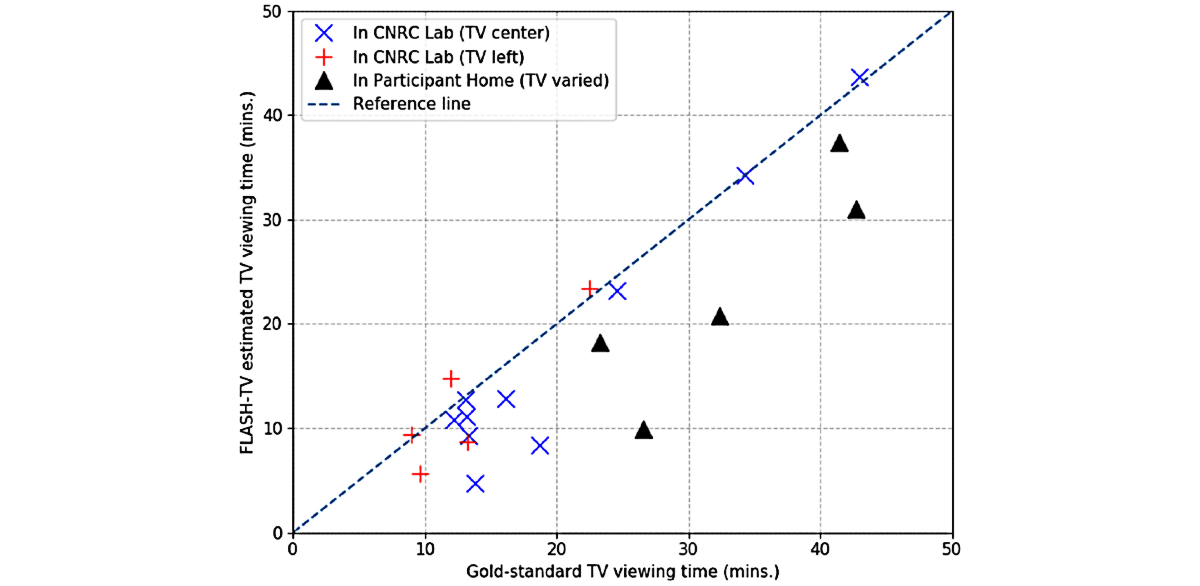
Scatter plot of gold standard television viewing time versus Family Level Assessment of Screen Use in the Home-Television (FLASH-TV) prediction. This plot compares gold standard television viewing time with FLASH-TV prediction for the 20 triads from our design tests. The television position for each data point is indicated in the legend. Most of our data points lie along the reference diagonal line (y = x) indicating the agreement between FLASH-TV and gold standard. The points below the diagonal indicate FLASH-TV underestimates (y < x) the television viewing time, whereas the points below the diagonal indicate that FLASH-TV overestimates (y > x) the viewing time. CNRC: Children’s Nutrition Research Center.

When implementing the 3 steps sequentially to estimate the target child’s television viewing time, the ICC was 0.725 when comparing the child’s estimated television viewing time per the FLASH three-step algorithm to the gold standard for total time, coded by staff (Table 7). The prevalence and bias-adjusted *κ* statistic was 0.728 (95% CI 0.727-0.729; *P*<.001) and the ICC comparing the number of frames identified as television viewing by the FLASH-TV with the human labelers was 0.401. The breakdown of correlations under different conditions is shown in Table 7. Figure 8 shows the comparison of television viewing time between FLASH-TV and the gold standard across 20 triads from our design tests.

A comparison of the television viewing time estimated by FLASH and the gold standard by race and ethnicity found that FLASH-TV underestimated television viewing time in all groups (Table 8).

**Table 7:** Family Level Assessment of Screen Use in the Home-Television (FLASH-TV) estimation of target child’s television viewing time during a 90-minute observation period.

Television position	FLASH-TV estimated television viewing time (minutes; range)	Gold standard television viewing time (minutes; range)	Television viewing frame by frame, *κ*^a^ (95% CI)	ICC^b^ of total television viewing time	ICC of television viewing frame by frame
Overall	17.47 (4.7-44.0)	21.72 (8.93-43.0)	0.728 (0.727-0.729)^c^	0.725	0.401
Center of wall (n=10)^d^	17.08 (4.7-44.0)	20.2 (12.2-43.0)	0.787 (0.786-0.788)^c^	0.717	0.428
Left corner of room (n=5)^d,e^	12.26 (5.5-23.3)	13.24 (8.93-22.5)	0.791 (0.789-0.793)^c^	0.762	0.392
In home, television position varied (n=5)^f^	23.5 (9.9-37.4)	33.3 (23.3-42.7)	0.499 (0.497-0.502)^c^	0.354	0.293

^a^Prevalence and bias-adjusted *κ* statistic.

^b^ICC: intraclass correlation.

^c^*P*<.001.

^d^Design tests 1 to 3 (in observation laboratory data collection).

^e^One family’s data from design tests 1 to 3 were obtained from a unique position (below television) and could not be used in gaze estimation training or testing.

^f^Design test 4 (in-home data collection).

**Table 8:** Exploration of race bias of Family Level Assessment of Screen Use in the Home-Television (FLASH-TV) television viewing estimates.

Child race and ethnicity	FLASH-TV estimated television viewing time (minutes), mean (SD)	Gold standard television viewing time (minutes), mean (SD)
Black^a^ (n=5)	7.62 (2.41)	13.26 (3.45)
Hispanic^b^ (n=5)	25.17 (10.46)	27.93 (9.95)
Non-Hispanic White (n=8)	20.0 (12.38)	25.16 (13.43)
Other^c^ (n=2)	12.06 (3.85)	12.63 (0.92)

^a^Black includes African American and Hispanic Black

^b^Hispanic includes Hispanic-White and Hispanic-unknown

^c^Other includes Asian and Hispanic-Filipino.

### Participants’ Thoughts About FLASH-TV

Most parents felt comfortable with or neutral toward having FLASH-TV in their home, especially as it would be helpful for them to see how much screen time their children obtained. Some participants were concerned with privacy: whether the camera would be recording all the time and who will have access to their data. Suggestions for improvement included having the ability to turn off the device at will, limiting to only video or audio data, and getting a breakdown about how their data are stored and processed.

## Discussion

### Principal Findings

FLASH-TV is being developed as an automated, objective assessment for measuring children’s screen use on stationary screens (eg, televisions, gaming systems, and computers) in the home, using deep-learning computer vision algorithms to process video data obtained from in front of a stationary screen. The FLASH-TV system estimates the time a child spends watching a specific screen by processing video data in three sequential steps for (1) face detection, (2) face verification of the target child, and (3) gaze detection of the target child. The findings from our study suggest that with further refinement, the FLASH-TV system can be a useful assessment tool for children’s viewing of large stationary screens. Output from FLASH-TV could be used in combination with other new assessment tools of other screen media use [11] to develop a composite of children’s screen media use across platforms.

### FLASH-TV Performance of Algorithms

The current version of FLASH-TV demonstrated high sensitivity for detecting faces when the child’s gaze was on the television (95.5%-97.9%) and poor-to-moderate PPV (52.5%-74.9%; Table 4). In developing the FLASH system, a low PPV was accepted for face detection to maximize the sensitivity (true positive rate) and keep false negatives low. This allows the FLASH-TV system to have a larger pool of images for the second step, face verification, which filters out all segments from step 1 that are not the target child, resulting in fewer false negatives from incorrect detection of the target child.

FLASH-TV demonstrated high (all >90%) accuracy, sensitivity, specificity, NPV, and PPV (Table 5) for correctly identifying the target child’s face under the condition that the child’s gaze was on the television, even when tested in the child’s home. The small differences in outcome metrics identified for face verification across visits by the same child were only present when the child was not watching television, except for the NPV. A significant difference in NPV between the 2 visits may indicate that FLASH-TV has some difficulty in identifying true negatives across days for a child. However, in this small sample, there were no differences in the outcome metrics by visit with the generalized linear models, which accounted for the correlation of data within a family. However, differences were found between families only for PPV. Qualitative assessment of the video data for face verification, where FLASH-TV performed the worst, suggests that the primary sources of error were when the child’s face was partially occluded or in dim lighting. Training the FLASH-TV face verification further in simulated darkened images of the child may help alleviate this moving forward.

The accuracy, specificity, and NPV were relatively high (>85%) for FLASH-TV when the gaze assessment was in the observation laboratory, compared with the home (>75%). However, the sensitivity was only moderate (73.2%-76.2%) and similar for each condition. PPV was higher and NPV was lower for in-home assessments. Data collected in the home, a free-living situation, are likely to contain more variability that will need to be addressed. In addition, qualitative assessment of gaze estimation suggests that the primary sources of error were low light, low resolution, and the child’s head orientation not being upright. Training the FLASH-TV gaze estimator on more varied data from different room configurations, different-sized televisions, and different locations of the television in the room should help address this moving forward. Simulating the current data with different head configurations and lighting conditions can provide additional training data to further refine the gaze estimator.

### FLASH-TV Estimation of Television Viewing

When combining the 3 sequential CNN visual-processing steps, FLASH-TV estimation of the child’s television viewing time was overall good (ICC for overall time watched television of 0.725 across conditions). However, a moderate ICC (0.401) was obtained when comparing the FLASH-TV system output for television viewing with the staff codes at the frame level. This suggests that sources of variability other than FLASH-TV or staff contribute to the estimation of a child’s television viewing time at the frame level, such as family unit, television position, or lighting during data collection. Therefore, FLASH-TV should not be used to assess gaze at extremely refined time increments defined at the frame level (1/30th of a second). In fact, researchers are unlikely to analyze children’s television viewing data at the second or minute level, reducing the impact of these unexplained sources of variability at the frame level. The next steps in refining the FLASH-TV output will include assessing whether smoothing the data into longer time epochs (eg, 5 s, 15 s, or 30 s) will smooth the variability caused by errors at the frame level to help improve the robustness of FLASH-TV in estimating children’s television viewing.

Qualitative analyses of the video data for the families where FLASH-TV performed the worst in estimating the child’s television watching time found that differences in the child’s viewing position were the most common challenge. Similar to other approaches using video images to measure children’s screen use [39], accuracy was impaired when children were reclining or laying on a couch or chair causing part or most of their face to be obscured. In these instances, the FLASH-TV often would not correctly identify the target child or their gaze, causing underestimation of television viewing. Training the FLASH-TV algorithms on larger data sets with the child reclining or laying down may help this.

Despite its current limitations, FLASH-TV is a significant improvement over current self- or parent-report methods that estimate how much time children spend watching television using gross categories. The ICC for the child’s total television viewing time for FLASH-TV was high (0.725), slightly better than that previously reported for television diaries (*r*=0.67), and much better than general estimates by parents (*r*=0.27) [18], which are commonly used in research [17]. Given how the data are collected within a family, our ICC estimates take into account nesting within the family unit, making them pragmatic and beneficial for powering future family-based studies. FLASH-TV also substantially decreases participant burden, which was noted to generate selection bias when using television diaries [18]. Other tools, such as TV Allowance, have been proposed as an objective assessment of television viewing among children [40]. The TV Allowance was developed for parents to limit their child’s access to television screens and required the child or parent to enter the child-specific code each time the child watches television. This may cause misclassification errors if the child is not watching the entire time the television is turned on or watches under another family member’s code. The TV Allowance only had a moderate correlation with parental estimates of television viewing in 4-to 7-year-old children [40] and preschool children [41]. In both studies, no comparison was made to the gold standard for direct or video-recorded observations. To our knowledge, the TV Allowance is no longer available for purchase. Forward-facing, wearable cameras automated to record images at frequent time intervals have also been investigated to estimate children’s screen use [39]. Such cameras appear to effectively capture images of screens (televisions, laptops, and smartphones) to which the child is exposed when the child is upright. However, similar to FLASH-TV, these cameras had problems when the child was laying down (capturing ceiling images instead). In addition, exposure to a screen does not mean that the child is attending to the programming. Furthermore, such cameras are dependent on the child wearing the camera, and wear time declines every evening over a 3-day study period from 78% to 51% of the evening time [39]. Placing the camera on the television instead, like FLASH-TV, places less burden on the child to complete the television watching assessment.

### Race Bias

Machine learning algorithms for face recognition and verification have come under scrutiny for not being as accurate across races, termed race bias [38]. Previous work has demonstrated that the source of race bias is related to both data-driven factors (eg, the representativeness of training data sets, the representativeness of the study population, and image conditions) and scenario-modeling factors (eg, thresholds used for face verification) [38]. Exploratory analyses of FLASH-TV suggest FLASH-TV underestimation of television viewing in all groups, but this may be greater among Black and non-Hispanic White children. However, the small sample size and variability in television viewing time between groups make statistical comparisons difficult at this stage. The design studies intentionally included a diverse sample of children to provide diverse training data to minimize the data-driven causes of race bias with FLASH-TV. Further refinement of FLASH-TV is needed, with continued attention to prevent the possibility of a race bias. If race bias occurs with larger sample sizes, approaches to mitigate race bias will be explored such as race-specific thresholds for face verification [38].

### Privacy Concerns

Assessments based on the collected images of a child’s varied surroundings raise concerns about privacy. Scientists using forward-facing wearable cameras have developed frameworks to manage the ethical considerations for capturing vast amounts of image data in various contexts [42,43]. The single location and context of the image data collected by FLASH-TV is different from that of wearable cameras. However, privacy issues remain. Some parents who participated in the design studies raised concerns about privacy issues with FLASH-TV. Once developed and deployed as a tool for measuring children’s television watching, the goal is to have FLASH-TV preserve privacy by only storing the processed output of the FLASH-TV machine learning algorithms and not storing the video data. This should address most of the parents’ concerns, but illustrating this to families before data collection may be important. However, until FLASH-TV has undergone further refinements and enhancements, studies require the video data to be stored to allow a gold standard for training the machine learning algorithms and to compare the FLASH-TV output. Such validation studies are critical to ensure the resulting system accurately captures a child’s television viewing behaviors and times [17] to allow for higher quality assessment in exposure studies and to assess the effect of television viewing reduction interventions.

### Limitations

To date, FLASH-TV has only been assessed during relatively short periods in task-based protocols to simulate scenarios of children’s typical screen use behaviors. Future studies will need to assess how robust FLASH-TV is in estimating a child’s screen use across multiple days. Most of the design tests conducted during the development of FLASH-TV were conducted in an observational laboratory. The location of the television in the room varied slightly across families, in addition to the participants’ location during each protocol. However, gaze estimation depends on the gaze vectors of the child. Therefore, the algorithms need to be trained on additional video data to capture a child’s gaze on a television screen in different positions in the room, resulting in different potential gaze vectors for the child. The sample of participants who took part in the design tests to help develop FLASH-TV were of varied race and ethnic backgrounds. FLASH-TV may not perform equally well for face detection and face verification across all families. Future analyses in larger, diverse data sets should evaluate whether child race, ethnicity, age, and similarity to sibling affect FLASH-TV time estimates for television viewing to ensure FLASH-TV works well across all groups of children. Finally, FLASH-TV does not assess content watched, or whether the child was active or sedentary while watching television or playing videogames. Future research should investigate the integration of FLASH-TV output with other data sources, such as accelerometer data, to better characterize the activity levels of children as they engage with screens.

### Conclusions

We have designed, developed, and performed initial design tests of FLASH-TV, the first-of-its-kind, quantitative, objective, automatic measurement tool for children’s television viewing. FLASH-TV offers a critical step forward in the assessment of children’s television viewing. Objective assessment of television viewing from FLASH-TV could be added to output from assessment tools of other screen platforms, for a composite measure of screen use among children to better inform research on the impact of screen use on children’s health and developmental outcomes.

## Appendix

Multimedia Appendix 1 Sample task-based protocol for design test 1.

## Abbreviations

CNN: convolutional neural network
FLASH-TV: Family Level Assessment of Screen Use in the Home-Television
ICC: intraclass correlation coefficient
NPV: negative predictive value
PPV: positive predictive value
