# Supplementary material for: An Objective System for Quantitative Assessment of Television Viewing Among Children (Family Level Assessment of Screen Use in the Home-Television): System Development Study
Source: JMIR Pediatr Parent. 2022 Mar 24;5(1):e33569. doi: 10.2196/33569 (PMC8990369; doi:10.2196/33569)
Supplement: Multimedia Appendix 1 [file pediatrics_v5i1e33569_app1.docx]

**Appendix 1: Sample task-based protocol for Design test 1.**

|  | **Design test 1: Child and parent TV watching protocol** | | | | | | | | |
| --- | --- | --- | --- | --- | --- | --- | --- | --- | --- |
| **Time (min)** | | **TV (on/off)** | **Tablet (on/off)** | **Child** | **Sibling** | **Parent** | **Room lighting*** | **Special Instructions** | **Device Variable Tested** |
| 0-3 | | ON | NA | (+) | (+) | (+) | Bright | Watch TV | Detect target child watching TV; Differentiate from parent and sibling |
| 3-6 | | ON | NA | (+) | (+) | (+) | Bright | Participants change location in room  Watch TV | Detect target child watching TV; Differentiate from parent and sibling |
| 6-9 | | OFF | NA | (+) | (+) | (+) | Bright | Provide toy basket  Do not watch TV | Detect target child not watching TV  Detect sibling and parent not watching TV |
| 9-12 | | ON | NA | (+) | (+) | (+) | Dim | Remove toy basket  Watch TV | Detect target child watching TV  Differentiate from parent and sibling |
| 12-15 | | ON | NA | (+) | (+) | (+) | Dim | Participants change location in room  Watch TV | Detect target child watching TV; Differentiate from parent and sibling |
| 15-18 | | OFF | NA | (+) | (+) | (+) | Dim | Participants change location in room  Provide toy basket  Do not watch TV | Detect target child not watching TV Detect sibling and parent not watching TV |
| Mobile Device Use | | | | | | | | | |
| 18-21 | | OFF | ON | (+) | (+) | (+) | Bright | Watch Tablet | Detect target child watching Tablet; Differentiate from parent and sibling |
| 21-24 | | OFF | ON | (+) | (+) | (+) | Bright | Participants change position  Watch Tablet | Detect target child watching Tablet; Differentiate from parent and sibling |
| 24-27 | | OFF | ON | (+) | (+) | (+) | Dim | Watch Tablet | Detect target child watching Tablet  Differentiate from parent and sibling |
| 27-30 | | OFF | ON | (+) | (+) | (+) | Dim | Participants change position Watch Tablet | Detect target child watching Tablet; Differentiate from parent and sibling |
| Free Play | | | | | | | | | |
| 33-45 | | ON | ON | (+) | (+) | (+) | Bright | Toy Basket present  Free play for child, sibling and parent with TV on, tablet and toys available | Identify what each subject is doing under bright light |
| 45-60 | | ON | ON | (+) | (+) | (+) | Dim | Toy Basket present  Free play for child, sibling and parent with TV on, tablet and toys available | Identify what each subject is doing under dim light |
|  | (+) person present in room participating in activity; (-) person NOT on couch participating in activity  *Room lighting: Bright- overhead and standing light on (~100 lux), Dim- only standing light on (~10 lux); Dark- all lights off (~1 lux) | | | | | | | | |

**Note:** Three participants from one family were included: parent and two siblings. One of the siblings was identified as the target child at the start of the study.
